# Colorimetric Quantification Methods for Peracetic Acid together with Hydrogen Peroxide for Water Disinfection Process Control

**DOI:** 10.3390/ijerph17134656

**Published:** 2020-06-28

**Authors:** Ravi Kumar Chhetri, Kamilla Marie Speht Kaarsholm, Henrik Rasmus Andersen

**Affiliations:** Department of Environmental Engineering, Technical University of Denmark, 2800 Kgs. Lyngby, Denmark; rakc@env.dtu.dk (R.K.C.); kmsh@env.dtu.dk (K.M.S.K.)

**Keywords:** peracetic acid, 2,2’-azino-bis(3-ethylbenzothiazoline-6-sulphonic acid (ABTS), N,N-diethyl-p-phenylenediamine (DPD), disinfection, hydrogen peroxide

## Abstract

Peracetic acid (PAA) water solutions is applied for disinfection of industry systems, food products and non-potable water. Commercially available peracetic acid is always supplied mixed with hydrogen peroxide (H_2_O_2_). H_2_O_2_ degrade slower than the peracetic acid which creates a need to quantify both peroxides separately to gauge the disinfection power of the solution and the residuals. Two combinations of colorimetric reactions are presented that allows simultaneous quantification at the mg·L^−1^ level used in disinfection liquids and water disinfection. The first dichromic reaction use titanium oxide oxalate (TiO-Ox) which only react with H_2_O_2_ followed by addition of N,N-diethyl-p-phenylenediamine with iodide (DPD/I^−^) and the concentrations are read by simultaneously measuring the absorbance at 400 and 515 nm. Limit of quantification (LOQ) and maximal concentration determined was 4.6 µg·L^−1^ and 2.5 mg·L^−1^ for PAA and 9.1 µg·L^−1^ and 5 mg·L^−1^ for H_2_O_2_. The two color reactions didn’t interfere with each other when the reagent addition was consecutive. Another combination of colorimetric reaction also used where TiO-Ox was used to first measure H_2_O_2_ at 400 nm, before addition of 2,2’-azino-bis(3-ethylbenzothiazoline-6-sulphonic acid (ABTS)) and reading the absorbance at 405 nm. ABTS changes the absorbance at 405 nm necessitating the two measurements be done separately. LOQ and maximal concentration determined using ABTS colorimetric assay was 42.5 µg·L^−1^ and 30 mg·L^−1^ for PAA and for titanium oxide oxalate colorimetric assay was 12.7 µg·L^−1^ and 75 mg·L^−1^ for H_2_O_2_. Both methods tested satisfactory in typical water samples (Tap, sea, lake, and biological treated sewage) spiked with peracetic acid and H_2_O_2_, separately.

## 1. Introduction

Disinfection is necessary to prohibit the spread of diseases and limiting the number of pathogenic organisms in the water industry. Various well known disinfectants are currently used in the water industry such as hypochlorite and chlorine dioxide [1], which could be used to reduce contamination by microorganisms. However, the toxic by-products of these compounds are of environmental concern [1,2,3,4,5,6,7,8,9]. Alternatively, peroxycarboxylic acids such as peracetic acid (PAA) has been used for disinfection. Furthermore, they do not generate toxic by-products [10,11]. Commercially available peroxycarboxylic acids are always available as a mixture of peroxycarboxylic acids, hydrogen peroxide (H_2_O_2_) and carboxylic acids.

PAA is a strong disinfectant with a wide spectrum of antimicrobial activity which was introduced to wastewater treatment approximately 30 years ago [12,13,14,15,16,17] and recently has been used to disinfect combined sewer overflows [11,18]. Commercial PAA is available as an acidic quaternary equilibrium mixture of PAA, H_2_O_2_, acetic acid, and water:

CH_3_COOH + H_2_O_2_ ⇌ CH_3_CO_3_H + H_2_O (1)

The residues after PAA use are acetic acid, H_2_O_2_, and water. Hydrogen peroxide tend to degrade slower than peroxycarboxylic acids [19] and it has a stringent discharge limit in relation to surface water.

To control the disinfection process, it is important to measure the exact concentration of disinfectant. The measured concentration of disinfectant is used to calculate and correlate the removal of microorganisms from disinfection process. Therefore, it is important to measure the residual concentration of both peroxycarboxylic acids and H_2_O_2_ when it is applied in the full scale disinfection and before it is discharged to the surface water to limit the adverse effect on aquatic life. 

Among various colorimetric assay, N,N-diethyl-p-phenylenediamine (DPD) with iodide (DPD/I)^−^ colorimetric assay [13,20,21] and ABTS (2,2’-azino-bis(3-ethylbenzothiazoline-6-sulphonic acid)) colorimetric assay [11,22], has been widely used for quantification of peroxycarboxylic acids and H_2_O_2_ using different reaction condition and reagents. Titanium oxide oxalate (TiO-Ox) color assay has been used for quantification of H_2_O_2_ [23]. Wagner et al., [19] have used ABTS colorimetric assay to measure concentration of PAA and H_2_O_2_ by measuring the concentration of total peroxycarboxylic acid and subtracting the concentration of H_2_O_2_ from the total peroxycarboxylic acid concentration. Similarly, Pedersen et al., [20] have used DPD colorimetric assay to measure peracetic acid and H_2_O_2_ separately using different reaction conditions and combining peroxidase enzyme. Domínguez-Henao et al., [24] have used two methods using DPD, iodine and ammonium molybdate as catalysis to determine PAA and total peroxide (PAA + H_2_O_2_). As of our knowledge, colorimetric assay that can simultaneously quantify peroxycarboxylic acids and H_2_O_2_ is not available. However, it is necessary when peroxycarboxylic acid is used for full-scale disinfection to control the dosing of peroxycarboxylic acid and discharge of residual peroxycarboxylic acid and H_2_O_2_. 

Therefore, the objective of this study was to develop a colorimetric assay which can quantify the peroxycarboxylic acids and H_2_O_2_ simultaneously. To do that, two combinations of two colorimetric assays TiO-Ox:DPD/I^−^ and TiO-Ox:ABTS were used. 

## 2. Materials and Methods 

### 2.1. Chemicals

All chemicals, unless stated otherwise, were purchased from Sigma-Aldrich (Brøndby, Denmark) and were of reagent grade. From peracetic acid (PAA) solution (30–40% *w*/*w* PAA and 5% *w*/*w* H_2_O_2_ of technical grade disinfectant), a working solution of 1 g·L^−1^ PAA was made, which was quantified daily by iodometric titration described below in Section 2.2. All solution were prepared in ultrapure water.

### 2.2. Sample Preparation and Analysis

Reaction time and reagents volume for color assay was optimized by measuring absorption spectra from 300 nm to 800 nm. A wavelength with high absorbance (peak) was selected from absorption spectra of oxidized TiO-Ox, DPD and ABTS to quantify H_2_O_2_ and PAA.

#### 2.2.1. ABTS Colorimetric Assay

Standard solution of peroxycarboxylic acid, H_2_O_2_ and samples were prepared in volumetric flasks. After dilution of the PAA stock solution to the concentration range from 0.5 mg·L^−1^ to 100 mg·L^−1^ in ultrapure water, 350 µL sample, 350 µL of 1M acetic acid (AA, pH 3.5) and 350 µL of ABTS (1 g·L^−1^ in ultrapure water) were added and color was allowed to develop for 10 min. The reaction products was observed photometrically at 405 nm in a 1.0 cm polypropylene cuvette [11]. Varian Cary 200 UV–Vis photometer was used to measure the absorbance.

#### 2.2.2. DPD/Iodide Colorimetric Assay

The DPD (N,N-dimethyl-p- phenylenediamine) color reagent was used to quantify PAA. The DPD reagent was prepared by mixing 70 mg EDTA and 385 mg DPD in 100 mL ultrapure water acidified by adding 25 mL of 0.5M sulfuric acid. Dilution of the PAA stock solution to the concentration range from 0.125 mg·L^−1^ to 3 mg·L^−1^ in ultrapure water was measured by adding 7 mL of sample, 100 µL AA (1 M, pH 5), 100 µL KI (70 g·L^−1^ in ultrapure water) and 100 µL DPD reagents in a glass beaker. After 1 min of reaction time, the reaction product was observed photometrically at 515 nm in a 1.0 cm polypropylene cuvette. 

#### 2.2.3. TiO-Ox Colorimetric Assay

H_2_O_2_ from sample was measured photometrically at 400 nm by adding 2 mL sample and 0.2 mL of potassium titanium oxide oxalate (50 g·L^−1^) in a 1.0 cm polypropylene cuvette after 1 min reaction time.

#### 2.2.4. TiO-Ox:DPD/I^−^ Colorimetric Assay 

PAA and H_2_O_2_ from sample were measured photometrically by adding 3.5 mL sample, 0.350 mL of potassium titanium oxide oxalate (50 g·L^−1^), 50 µL AA (1 M, pH 5), 50 µL KI (70 g·L^−1^ in ultrapure water) and 50 µL DPD reagents in a glass beaker. After 1 min of reaction time, the reaction product was observed photometrically at 400 nm for H_2_O_2_ and 515 nm for PAA in a 1.0 cm polypropylene cuvette.

#### 2.2.5. Samples

Secondary treated wastewater effluent was collected from Lundtofte WWTP, Denmark. Sea water was collected from Øresund Sea, lake water was collected from Gentofte Lake and non-chlorinated tap water. Samples were spiked with different concentration of PAA and concentration of PAA were quantified using TiO-Ox and DPD/I^−^ and TiO-Ox and ABTS color assay described above. 

#### 2.2.6. Iodometric Titration

The two step titration was used to determine the H_2_O_2_ and peroxycarboxyclic acid based on the procedures described originally by [25]: a 100–200 mg of samples was accurately weighed into the titration vessel containing 10 mL of 5% H_2_SO_4_ solution. One piece of ice was added to maintain the temperature of the aliquot solution under 10 °C. An amount of 40 mL H_2_SO_4_ solution and 3 drops of ferroin indicator were added and the sample was titrated with 0.1 N ammonium cerium sulphate solution from orange to light blue. To determine the peroxycarboxylic acids concentration, 5 mL of the 10% KI solution, 3 drops of the ammonium heptamolybdate solution and 1 mL of the starch solution were added in the aliquot solution from which H_2_O_2_ was titrated. The liberated iodine was titrated with 0.1 N Na_2_S_2_O_3_ solution from dark brown color to orange. The concentration of H_2_O_2_ and PAA determined from titration was used to measure the dilution of the PAA and H_2_O_2_ stock solution for colorimetric assay.

### 2.3. Limit of Detection and Quantification of Analytical Methods

The calibration curve was acquired by linear regression analysis. Blank and lowest concentration was measured in a seven replicate to measure the limit of detection (LOD) and the limit of quantification (LOQ). The LOD was calculated as 3 times the standard deviation of the blank and the LOQ as 10 times of the standard deviation of the blank. Sample absorbance was corrected for reagent blanks and concentrations calculated using a standard curve slope. The tested concentration ranges were (i) for DPD/I^−^ PAA 0.125–10 mg·L^−1^ PAA, (ii) for ABTS 0.125–50 mg·L^−1^ PAA, and (iii) for TiO-Ox 0.025–100 mg·L^−1^ H_2_O_2_, (iv) for TiO-Ox:DPD/I^−^ 0.125–5 mg·L^−1^ H_2_O_2_ and 0.125–10 mg·L^−1^ PAA.

## 3. Results and Discussion

### 3.1. Principle

DPD reagent together with potassium iodide is widely used to quantify chlorine species, PAA and H_2_O_2_ in water samples from different sources [1,20]. The purposed colorimetric assay (Method 1) with DPD/I^−^ and titanium oxide oxalate measures the concentration of both peroxycarboxylic acid and H_2_O_2_ in single step at two different wavelengths which makes it easy to quantify both PAA and H_2_O_2_ together without interference from each other. 

**Figure 1:**
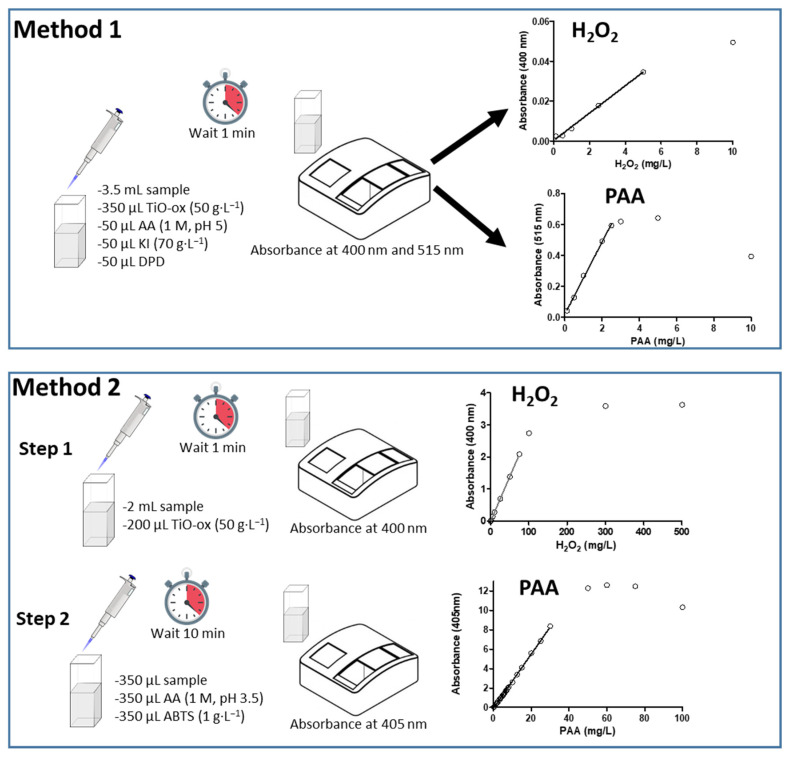
Schematic diagram showing the steps of the two methods used for quantification of peracetic acid (PAA) and hydrogen peroxide (H_2_O_2_): (TiO-Ox: titanium oxide oxalate; AA: Acetic acid; KI: potassium iodide; DPD: N,N-dimethyl-p- phenylenediamine; ABTS: 2,2’-azino-bis(3-ethylbenzothiazoline-6-sulphonic acid).

ABTS colorimetric assay was used together with TiO-Ox to quantify the PAA and H_2_O_2_ in two steps (Method 2). In the first step, TiO-Ox react with H_2_O_2_ that is present in PAA to make a complex compounds which can quantified at 400 nm whilst in Step 2, PAA can oxidize colorless ABTS to green color ABTS^•+^ which can quantified spectrophotometrically at 405 nm (Figure 1). 

### 3.2. Selectivity of Colorimetric Reactions

#### 3.2.1. Hydrogen Peroxide Reaction with TiO-Ox, DPD and ABTS

**Figure 2:**
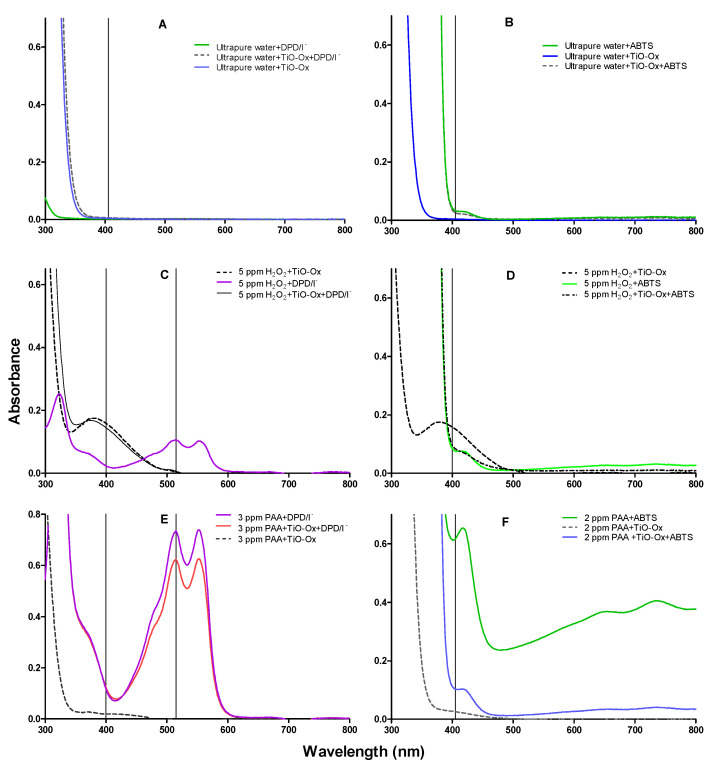
Absorption spectra of oxidized colorimetric reagents; (**A**) absorption spectra of DPD/I^−^, [TiO-Ox·DPD/I^−^] and TiO-Ox mixed in ultrapure water to measure reagents background color and cross reactivity between the reagents, (**B**) absorption spectra of ABTS, [TiO-Ox·ABTS] and TiO-Ox mixed in ultrapure water to measure reagents background color and cross reactivity between the reagents, (**C**) absorption spectra of DPD·^+^ and [TiO-Ox·H_2_O_2_] obtained by oxidation with 5 mg·L^−1^ H_2_O_2_, (**D**) absorption spectra of ABTS^·+^, [TiO-Ox·H_2_O_2_] and [TiO-Ox· ABTS·^+^·H_2_O_2_] obtained by oxidation with 5 mg·L^−1^ H_2_O_2_, (**E**) absorption spectra of DPD·^+^, and [TiO-Ox· DPD·^+^·PAA] obtained by oxidation with 3 mg·L^−1^ PAA. Spectra after color reaction with 3 mg·L^−1^ PAA, DPD/I^−^; PAA, TiO-Ox, DPD/I^−^ and PAA, TiO-Ox. All color reaction was maintained at pH 5 in presence of KI (70 g·L^−1^) and (**F**) absorption spectra of ABTS^·+^, [TiO-Ox·PAA] and [TiO-Ox· ABTS^·+^·PAA] obtained by oxidation with 2 mg·L^−1^ PAA.

Reagents used in DPD/I^−^, ABTS and TiO-Ox colorimetric assay were mixed with ultrapure water and absorption spectra were measured in the same condition as it was measured for colorimetric assay of ABTS and DPD/I^−^ mentioned in Section 2.2. This was done to check the cross reaction of reagents itself without adding oxidants. It was observed that reagents; DPD/I^−^ and TiO-Ox or ABTS and TiO-Ox does not react to each other and does not develop the color which can be quantified spectrophotometrically (Figure 2A,B). 

When H_2_O_2_ reacted with TiO-Ox, the yellow colored titanium peroxide complex was formed which can be measured spectrophotometrically at 400 nm (Figure 2C). Furthermore, when H_2_O_2_ reacted with DPD/I^−^ reagents buffered at pH 5, the pink colored oxidized form of DPD (DPD^+^) was formed (Figure 2C). The DPD·^+^ has an absorption peak at 515 nm, which usually is used for quantification. However, when mixing the formed titanium peroxide complex with the DPD/I^−^ reagents at pH 5, no peak was observed at 515 nm (Figure 2C). Thus, it was observed that complex formed from the reaction of TiO-Ox and H_2_O_2_ does not react with DPD/I^−^. This makes combined colorimetric reaction of TiO-Ox and DPD/I^−^ selective to measure H_2_O_2_ and peroxycarboxylic acid simultaneously. Furthermore, no change in the absorption spectra was observed when the titanium peroxide complex was mixed with ABTS compared with the absorption spectra of H_2_O_2_ reacted with ABTS at 400 nm (Figure 2D). Moreover, the absorbance of TiO-Ox:H_2_O_2_ complex at 400 nm was higher than the absorbance of ABTS oxidized by H_2_O_2_ (Figure 2D) since TiO-Ox colorimetric assay was selective for H_2_O_2_.

#### 3.2.2. Peracetic Acid Reaction with TiO-Ox

**Figure 3:**
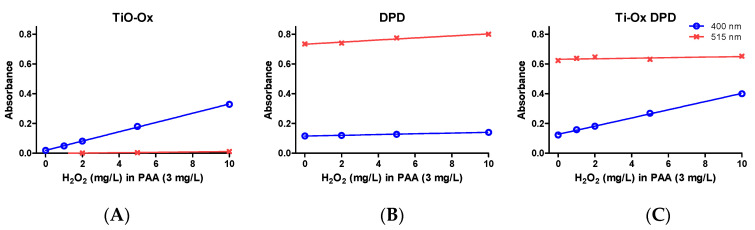
Absorbance of oxidized colorimetric reagents; absorbance of TiO-Ox obtained by oxidation with 0, 1, 2, 5 and 10 mg·L^−1^ H_2_O_2_ spiked in 3 mg·L^−1^ PAA (**A**); absorbance of DPD^•+^ obtained by oxidation with 0, 1, 2, 5 and 10 mg·L^−1^ H_2_O_2_ spiked in 3 mg·L^−1^ PAA (**B**) and absorbance of TiO-Ox:DPD·^+^ obtained by oxidation with 0, 1, 2, 5 and 10 mg·L^−1^ H_2_O_2_ spiked in 3 mg·L^−1^ PAA (**C**). Absorbance were recorded at 400 nm and 515 nm. Error bars indicating standard deviation (*n* = 3) were always smaller than the symbol representing the averages.

Commercial solutions of PAA contains different ratio of PAA:H_2_O_2_ in equilibrium. When TiO-Ox was mixed with 3 mg·L^−1^ commercial PAA, it reacted with H_2_O_2_ that was present in the commercial PAA solution resulting in fainted yellow color and high absorbance at 400 nm (Figure 2E). To check the selective reaction of TiO-Ox and H_2_O_2_, absorbance recorded at 400 nm was converted to the H_2_O_2_ concentration using calibration curve and same concentration of H_2_O_2_ was spiked to ultrapure water and quantified using TiO-Ox reagents. Same absorbance at 400 nm was observed when TiO-Ox was mixed with 3 mg·L^−1^ commercial PAA in ultrapure water spiked with H_2_O_2_ solution (Figure 3A). Moreover, when 1, 2, 5 and 10 mg·L^−1^ H_2_O_2_ was spiked in 3 mg·L^−1^ PAA and measured with TiO-Ox, the absorbance at 400 nm increased with increasing H_2_O_2_ concentration (Figure 3B). This shows TiO-Ox does not react with PAA but can selectively react with H_2_O_2_. 

#### 3.2.3. Peracetic Acid Reaction with DPD/I^−^

Experiments were performed to investigate whether PAA reacts with DPD/I^−^ buffered at pH 5 with and without presence of TiO-Ox. The oxidation of colorless DPD to its pink product DPD·^+^ by 3 mg·L^−1^ PAA at pH 5 in presence of iodide was observed and the absorption spectrum from 300 nm to 800 nm was measured (Figure 2E). An absorbance peak was observed at 515 nm which usually is used for quantification of DPD·^+^. In another experiment, TiO-Ox was added prior to DPD/I^−^, to investigate the interference of TiO-Ox on oxidation of DPD by PAA. The absorption spectrum of DPD/I^−^ and PAA in presence of TiO-Ox showed that DPD was oxidized by PAA and pink color was developed (Figure 2E). However, less color development was observed since the H_2_O_2_ present in the PAA mixture reacted with TiO-Ox and not with DPD. A slight increase in absorbance at 400 nm was observed which was due to the reaction of TiO-Ox with H_2_O_2_.

The selectivity of the reaction was tested by measuring a 3 mg·L^−1^ PAA sample spiked with 1, 2, 5 and 10 mg·L^−1^ H_2_O_2_ and absorbance was measured at 400 nm and 515 nm with TiO-Ox, DPD/I^−^ and TiO-Ox:DPD/I^−^ color assays. When TiO-Ox color assay was tested, absorbance at 400 nm increased with increasing concentration of H_2_O_2_ as mentioned in the Section 3.2.2 while absorbance at 515 nm was close to zero (Figure 3A). When 3 mg·L^−1^ PAA spiked with H_2_O_2_ were quantified with DPD/I^−^ color assay, a small but fixed peak was observed at 400 nm with almost no effect of increasing H_2_O_2_ concentration while at 515 nm the absorbance increased with increasing concentration of H_2_O_2_ (Figure 3B). When 3 mg·L^−1^ PAA spiked with H_2_O_2_ were quantified with TiO-Ox:DPD/I^−^, the absorbance at 400 nm increased however, this was not the case at 515 nm (Figure 3C). The quantification of PAA was shown not to be biased by the presence of H_2_O_2_, as addition of 1, 2, 5 and 10 mg·L^−1^ H_2_O_2_ in 3 mg·L^−1^ PAA did not affect the absorbance at 515 nm (DPD·^+^) but resulted in increased absorption at 400 nm (titanium peroxide complex). Thus, when H_2_O_2_ reacts with TiO-Ox to form titanium peroxide complex, it will not react further with DPD/I^−^ color reagent under the given condition. 

Therefore, it was shown that DPD/I^−^ for PAA quantification was not interfered by presence of TiO-Ox and TiO-Ox:DPD/I^−^ color assay can be used selectively to measure PAA and H_2_O_2_.

#### 3.2.4. Peracetic Acid Reaction with ABTS 

Colorless ABTS was oxidized to green colored ABTS·^+^ by PAA at pH 3.5 (Figure 2F) which was also observed in our previous study [11]. When three different concentration of PAA, 1 mg·L^−1^, 3 mg·L^−1^ and 5 mg·L^−1^, was mixed with ABTS, absorbance at 405 nm increased gradually (see SI, Figure S1). An experiment was performed to investigate whether presence of TiO-Ox interfere with the color development of ABTS when oxidized by PAA. Absorbance of ABTS·^+^ at 405 nm was higher than the absorbance of ABTS·^+^ in the presence of TiO-Ox and thus TiO-Ox interferes the reaction of ABTS with PAA. To eliminate the interference of TiO-Ox on ABTS quantification, the two-step quantification method was applied where absorbance of TiO-Ox and PAA was measured in one cuvette and absorbance of ABTS^•+^ oxidized by PAA was measured in a second cuvette.

### 3.3. Method Characterization

**Figure 4:**
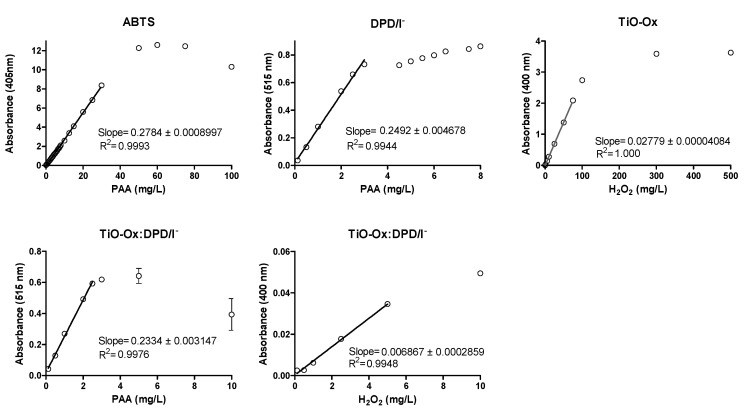
Calibration curve of PAA and H_2_O_2_ using ABTS, DPD/I^−^, TiO-Ox:DPD/I^−^ and TiO-Ox as color reagents, respectively. Error bars indicating standard deviation (*n* = 3) are smaller than the symbol representing the averages for most concentrations. Note: different scales on the primary axes.

Calibration curve was made for PAA and H_2_O_2_ using different concentration of oxidants by using DPD/I^−^, ABTS, TiO-Ox and TiO-Ox:DPD/I^−^ colorimetric assay as described in Section 2.2 (Figure 4 and Table 1). LOD and LOQ for ABTS colorimetric assay for PAA was 0.0425 mg·L^−1^and 0.0500 mg·L^−1^, respectively.

From the ABTS colorimetric assay direct measurement of PAA was done up to 30 mg·L^−1^ with R-square 0.9993. LOQ and LOD for DPD/I^−^ colorimetric assay for PAA was 0.0015 mg·L^−1^and 0.0025 mg·L^−1^, respectively. Direct measurement of PAA was done up to 3 mg·L^−1^ by using DPD/I^−^ colorimetric assay with R-square 0.9944. LOQ and LOD for TiO-Ox:DPD/I^−^ colorimetric assay for PAA was 0.0024 mg·L^−1^and 0.0046 mg·L^−1^, respectively. Direct measurement of PAA was done up to 2.5 mg·L^−1^ by using TiO-Ox:DPD/I^−^ colorimetric assay with R-square 0.9976. Moreover, a calibration curve of H_2_O_2_ was made from the absorbance recorded at 400 nm using TiO-Ox:DPD/I^−^ colorimetric assay when calibration curve was made for PAA. This was done by subtracting the absorbance of TiO-Ox:DPD·^+^ with DPD·^+^ at 400 nm and from this method direct measurement of H_2_O_2_ was done up to 5 mg·L^−1^ with R-square 0.9948. LOD for TiO-Ox:DPD/I^−^ colorimetric assay for H_2_O_2_ was 0.0043 mg·L^−1^ and 0.0091 mg·L^−1^, respectively. Thus, TiO-Ox:DPD/I^−^ colorimetric assay is suitable for the water industry that demands low concentration of disinfectant whilst ABTS colorimetric assay was suitable for the water industry that demand higher disinfectant dose. LOD and LOQ for TiO-Ox and H_2_O_2_ was 0.0080 mg·L^−1^and 0.0127 mg·L^−1^, respectively. Direct measurement of H_2_O_2_ was done up to 75 mg·L^−1^ with R-square 1.00.

### 3.4. Suitability of Colorimetric Assay for Different Samples

**Figure 5:**
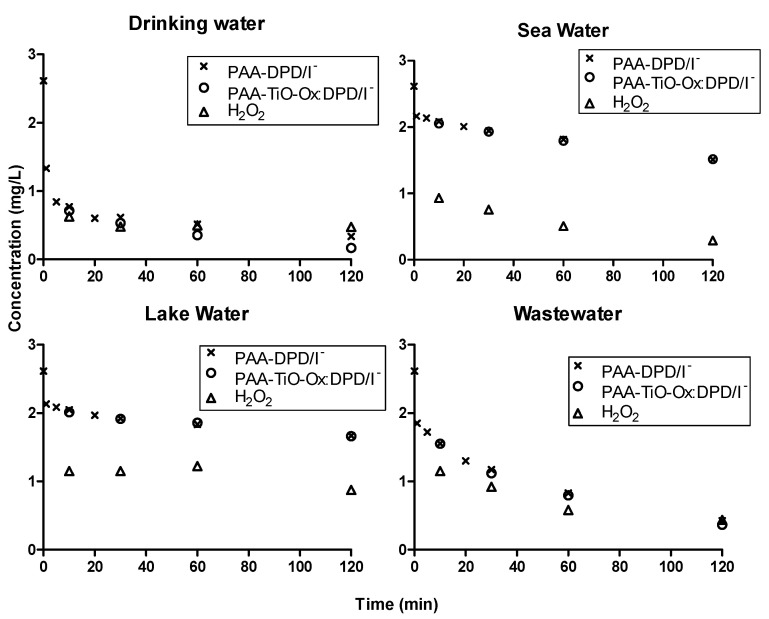
Concentration profiles of PAA and H_2_O_2_ in drinking water, sea water, lake water and wastewater effluent quantified using DPD/I^−^ and TiO-Ox:DPD/I^−^ colorimetric assay when spiked with 2.6 mg·L^−1^ PAA.

**Figure 6:**
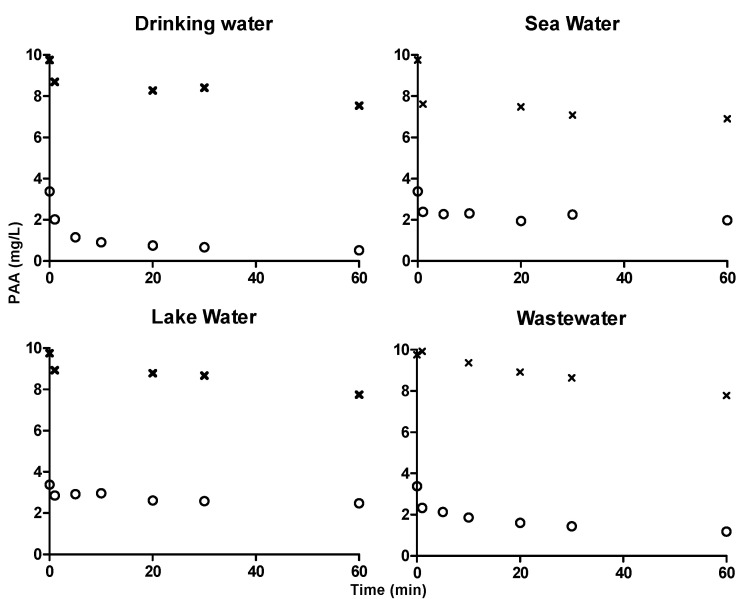
Concentration profiles of PAA in drinking water, seawater, lake water and wastewater quantified using ABTS colorimetric assay. ‘x’ symbol in the graphs represents the concentration profile of 10 mg·L^−1^ PAA and ‘o’ symbol in the graphs represents the concentration profile of 3 mg·L^−1^ PAA.

The suitability of colorimetric assay in different samples with different water matrices were studied by measuring concentration profiles of PAA using DPD/I^−^, TiO-Ox:DPD/I^−^ and ABTS color assay (Figure 5 and Figure 6). DPD/I^−^ and TiO-Ox:DPD/I^−^ color assay was used to study the degradation of 2.6 mg·L^−1^ PAA for 120 minutes in drinking water, sea water, lake water and wastewater effluent. Slow degradation of PAA was observed in lake water and sea water, while rapid degradation of PAA was observed in wastewater effluent and drinking water. The different degradation of PAA were due to the samples having different water matrices. The concentration of PAA quantified using DPD/I^−^ and TiO-Ox:DPD/I^−^ colorimetric assay was identical (Figure 5). Moreover, with TiO-Ox:DPD/I^−^ colorimetric assay H_2_O_2_ was also quantification which is important when applying PAA for disinfection. 

ABTS colorimetric assay was used to quantify 3 mg·L^−1^ and 10 mg·L^−1^ PAA in drinking water, sea water, lake water and wastewater for 60 min (Figure 6). Degradation of PAA in sea water and lake water were slow and similar to that was quantified using DPD/I^−^ and TiO-Ox:DPD/I^−^ color assay. Hence, ABTS and DPD/I^−^ color assay can be used to quantify PAA and TiO-Ox:DPD/I^−^ color assay can be used to quantify PAA and H_2_O_2_ simultaneously in the samples with different characteristics.

## 4. Conclusions

An optimized combination of two colorimetric reactions are presented that allows simultaneous quantification of H_2_O_2_ and a peroxycarboxylic acid at the mg·L^−1^ level when it is applied in water for disinfection purposes. H_2_O_2_ was quantified by adding titanium oxide oxalate (TiO-Ox) followed by addition of DPD/I^−^ and allow color development so both concentrations could be determined simultaneously in one cuvette. In another combination of colorimetric reaction, H_2_O_2_ was quantified by TiO-Ox from the mixture of peroxycarboxylic acids in first step followed by quantification of peroxycarboxylic acids by addition of ABTS in second step in separate vials. 

LOD and LOQ of optimized combination of two colorimetric reactions were measured followed by measuring maximum linear concentration of peroxycarboxylic acids and H_2_O_2_. LOD and LOQ of ABTS colorimetric assay was 42.5 µg·L^−1^and 50 µg·L^−1^, DPD/I^−^ colorimetric assay was 1.5 µg·L^−1^and 2.5 µg·L^−1^, TiO-Ox:DPD/I^−^ colorimetric assay was 2.4 µg·L^−1^and 4.6 µg·L^−1^ and for titanium oxide oxalate colorimetric assay was 8.0 µg·L^−1^and 12.7 µg·L^−1^, respectively. Direct measurement of PAA using ABTS was up to 30 mg·L^−1^ for PAA, up to 3 mg·L^−1^ PAA by using DPD/I^−^ colorimetric assay and up to 2.5 mg·L^−1^ PAA and 5 mg·L^−1^ H_2_O_2_ by using TiO-Ox:DPD/I^−^ colorimetric assay. By using titanium oxide oxalate colorimetric assay 80 mg·L^−1^, H_2_O_2_ was measured directly. 

Suitability of the colorimetric method for PAA and H_2_O_2_ was tested by spiking peracetic acid to the water samples from typical applications; tap water, lake water, seawater, and biological treated municipal wastewater. The concentration of PAA and H_2_O_2_ were quantified over time by applying combination of two colorimetric assay; TiO-Ox:DPD/I^−^ and TiO-Ox:ABTS.

## Figures and Tables

**Table 1:** Limit of detection, limit of quantification and maximum linearity of ABTS, DPD/I^−^, TiO-Ox:DPD/I^−^ and TiO-Ox colorimetric assay.

	ABTS Assay (mg·L^−1^)	DPD/I^−^ Assay (mg·L^−1^)	TiO-Ox:DPD/I^−^ Assay-PAA (mg·L^−1^)	TiO-Ox:DPD/I^−^ Assay-H_2_O_2_ (mg·L^−1^)	TiO-Ox Assay (mg·L^−1^)
LOD	0.0425	0.0015	0.0024	0.0043	0.0080
LOQ	0.0500	0.0025	0.0046	0.0091	0.0127
Maximum linearity	30	3	2.5	5	75

## Supplementary Materials

The following are available online at https://www.mdpi.com/1660-4601/17/13/4656/s1, Figure S1: Absorption spectra of oxidized ABTS mixed with 1, 3 and 5 mg·L-1 PAA in ultrapure water (left), Absorption spectra of oxidized TiO-Ox mixed with 1, 3 and 5 mg·L-1 PAA in ultrapure water (right).
