# Supplementary material for: Colorimetric Quantification Methods for Peracetic Acid together with Hydrogen Peroxide for Water Disinfection Process Control
Source: Int J Environ Res Public Health. 2020 Jun 28;17(13):4656. doi: 10.3390/ijerph17134656 (PMC7369690; doi:10.3390/ijerph17134656)
Supplement: Supplementary file 1 [file ijerph-17-04656-s001.pdf]

Supporting information

Colorimetric quantification methods for peracetic acid together with hydrogen peroxide for water disinfection control

Ravi Kumar Chhetri[a], Kamilla M.S. Kaarsholm[a], Henrik Rasmus Andersen[a1]

[a]Department of Environmental Engineering, Technical University of Denmark, Bygningstorvet, Building 115, 2800 Kgs. Lyngby, Denmark. [1]Corresponding author: Henrik@ndersen.net

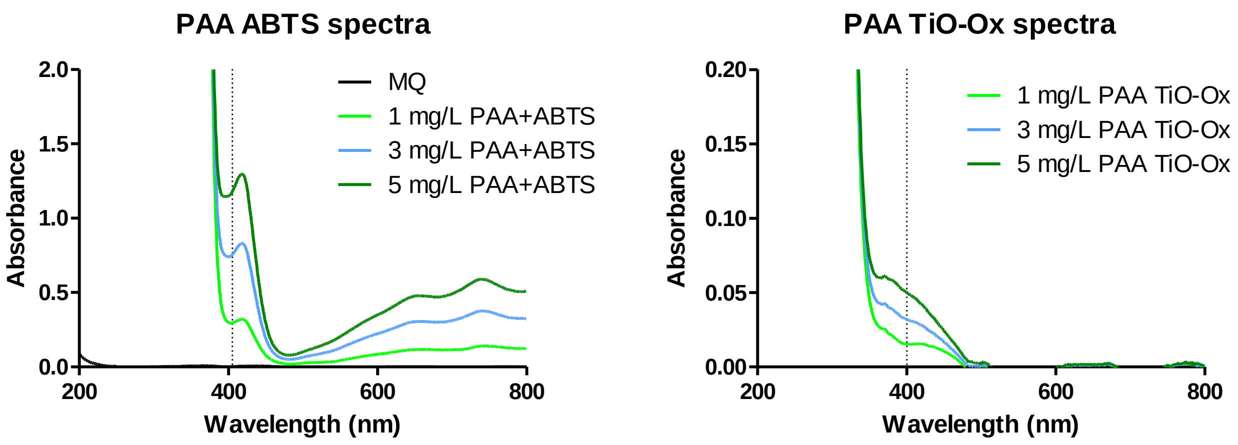

Figure S1: Absorption spectra of oxidized ABTS mixed with 1, 3 and 5 mg·L^{-1} PAA in ultrapure water (left), Absorption spectra of oxidized TiO-Ox mixed with 1, 3 and 5 mg·L^{-1} PAA in ultrapure water (right).
